# The impact of COVID-19 on dental care in New York State and Georgia

**DOI:** 10.1038/s41415-023-5458-9

**Published:** 2023-01-24

**Authors:** Caroline Puskas, Stephen S. Morse

**Affiliations:** 41415113297001 Dental Student, Columbia University College of Dental Medicine, 622 West 168th Street, New York, NY 10032, USA; 41415113297002 Professor, Department of Epidemiology, Columbia University Mailman School of Public Health, 722 West 168th Street, #1504, New York, NY 10032, USA

## Abstract

**Introduction** During the COVID-19 pandemic, there was an unprecedented and forced closure of dental offices worldwide. As American state recommendations differed considerably during this period, this research strives to better define the effects of this pause on dental care.

**Materials and methods** A 16-question Qualtrics survey was sent to the membership of the New York State Dental Association (NYSDA) and Georgia Dental Association (GDA). Licenced, actively practising dental members of the NYSDA and GDA (n = 680) answered questions about their practice demographics, appointment cancellations, reopening times and the volume of individual dental procedures performed from 1 March through to 1 August 2020, compared to the same five-month period in 2019.

**Results** Demographic characteristics of respondent NYSDA and GDA members were statistically similar. Nonetheless, NYSDA members reported significantly larger decreases in provision of all types of dental procedures, except for antibiotic prescription, including prophylaxis, elective care, emergency dental care and speciality procedures.

**Discussion and conclusions** All dental procedures declined significantly during the COVID-19 pandemic, with greater decrease in New York than in Georgia. This study raises concerns about the negative impact of the pandemic on oral public health and mandates both further research and clinical strategies to mitigate against this future risk.

**Supplementary Information:**

Zusatzmaterial online: Zu diesem Beitrag sind unter 10.1038/s41415-023-5458-9 für autorisierte Leser zusätzliche Dateien abrufbar.

## Key points

- Informs readers of the development of key issues of dentistry and oral public health as a result of the COVID-19 pandemic.
- Provides a better understanding of what dental procedures did and did not occur during the COVID-19 pandemic in the states of New York and Georgia.
- Investigates the impact of fear of COVID-19 exposure, by the patient and provider, on dental care.
- Functions as a base for future investigations of the COVID-19 pandemic and its effect on oral public health.

**Supplementary Information:**

Zusatzmaterial online: Zu diesem Beitrag sind unter 10.1038/s41415-023-5458-9 für autorisierte Leser zusätzliche Dateien abrufbar.

## Introduction

During the onset of the COVID-19 pandemic, there was an unprecedented and forced closure of dental offices in the USA. While much of the initial dental research in relation to the COVID-19 pandemic pertains to infection control and guidance on dental precautions, the specific dental care provided in the USA during the COVID-19 pandemic has more recently undergone investigation.^1^

Dentistry includes many aerosol generating procedures that are associated with increased transmission of respiratory infection.^2^ Thus, it has been hypothesised that dentists are at increased risk of occupational exposure to the novel coronavirus, as COVID-19 is spread via respiratory droplets and is detectable in saliva.^3^ Interestingly, research has also found that reported rates of COVID-19 infection among dental professionals is not significantly different from the general population.^4^

While the American Dental Association (ADA) and Centers for Disease Control and Prevention recommended that American dentists should postpone elective procedures, surgeries, and non-urgent dental visits, solely prioritising urgent and emergency visits, individual state governors mandated the specific dates on which states could re-open their dental practices.^5^ State regulatory bodies mandated that elective care be paused and permission for dental practices to reopen to their full scope of services varied from state to state.^6^

These state guidelines and national recommendations were widely communicated but the degree to which dentists complied with the national recommendations and state mandates remains unknown. Further, variable interpretation of what constituted emergency dental care is suspected.

As the first author of this research was raised in Georgia and attends dental school in New York, those two states with varying COVID-19 experiences were primarily analysed. Governor Kemp of Georgia permitted the full reopening of dental offices on 1 May 2020 with adherence to the ADA's guidelines of transmission minimisation and personal protective equipment. In comparison, Governor Cuomo of New York State announced approval of state-wide opening of dental offices for regular dental care one month later, on 1 June 2020.^6^

Beyond state regulation of dental practice reopening dates, fear of COVID-19 exposure had a significant impact on patients' willingness to seek dental care and dentists' willingness to reopen practices to non-urgent dental care. Similarly, during the 2003 Severe Acute Respiratory Syndrome (SARS) epidemic, patients' access to care was compromised by their diminished care-seeking behaviour due to fear of SARS.^7^ The present study investigated the shift in care-seeking behaviour during the COVID-19 pandemic, as was seen previously in the SARS epidemic, by inquiring what proportion of cancelled/rescheduled appointments were due to patients' fear of COVID-19 exposure in the dental office.

The objective of this research was to quantify dental procedures provided in the states of New York and Georgia during the COVID-19 pandemic compared to the year prior and to investigate how fear of COVID-19 exposure contributed to appointment cancellations and deferrals when dental practices were reopened.

Coincident with present research, a similar survey of dental practitioners in Brazil was published.^8^ The web-based survey sent to Brazilian dentists found that despite the lockdown recommendations, 83.8% of the dentists reported their patients continued to seek elective dental care, including prophylaxis and preventive procedures during the designated two-week response time between 5 May and 20 May 2020.^8^ Similarly, our study attempts to quantify the impact of the COVID-19 pandemic on specific dental procedures provided during the peak five-month period of the pandemic, but in the USA.

## Materials and methods

A 16-question Qualtrics survey (Qualtrics Software Company, Provo, UT, USA) was designed and tested with a mixed pilot group of 13 dentists in the New York State Dental Association (NYSDA) and Georgia Dental Association (GDA) who provided feedback on the survey. The survey instrument and its planned administration were reviewed by the Institutional Review Board of the Human Research Protection Office of Columbia University and was approved as exempt based on the anonymity and minimal risk entailed in the research. The revised Qualtrics survey was emailed to the membership of the NYSDA and GDA from the respective association headquarters, emphasising the voluntary and anonymous nature of the survey with no sensitive or identifiable information. New York- and Georgia-based dentists were invited to participate in the survey if they were members of the NYSDA or GDA and licenced, actively practising dentists of any dental speciality. In total, 10,005 NYSDA members received an invitation to participate in the survey on 16 September 2020, while 3,559 GDA members received an invitation to participate in the survey on 28 September 2020. As 506 NYSDA members and 174 GDA members responded, the response rates were 5.06% and 4.89%, respectively. Study participants consented to respond to the voluntary and anonymous email survey tool and were clearly informed of the purpose of the survey and the intent to analyse and publish the data returned in an aggregate, de-identified format. The complete 16-question Qualtrics survey is in the online Supplementary Information.

Demographic questions requested the zip code of the dental office, how many years the respondent dentist has been in practice, the number of dentists practising at the primary site and whether the dentist(s) accept(s) Medicaid. Subsequent questions inquired into how the dental practice's cancellation rates changed during the COVID-19 pandemic compared to the year prior, and an estimate of how much of that change was accounted for by cancellations due to fear of COVID-19 exposure. The final questions of the survey sought to quantify the change in the amount of individual dental procedures provided for patients during the COVID-19 pandemic, including dental prophylaxis, direct restorations, crowns, implants, extractions, endodontic treatment, orthodontic treatment, periodontal treatment and antibiotic prescriptions.

**Fig. 1:**
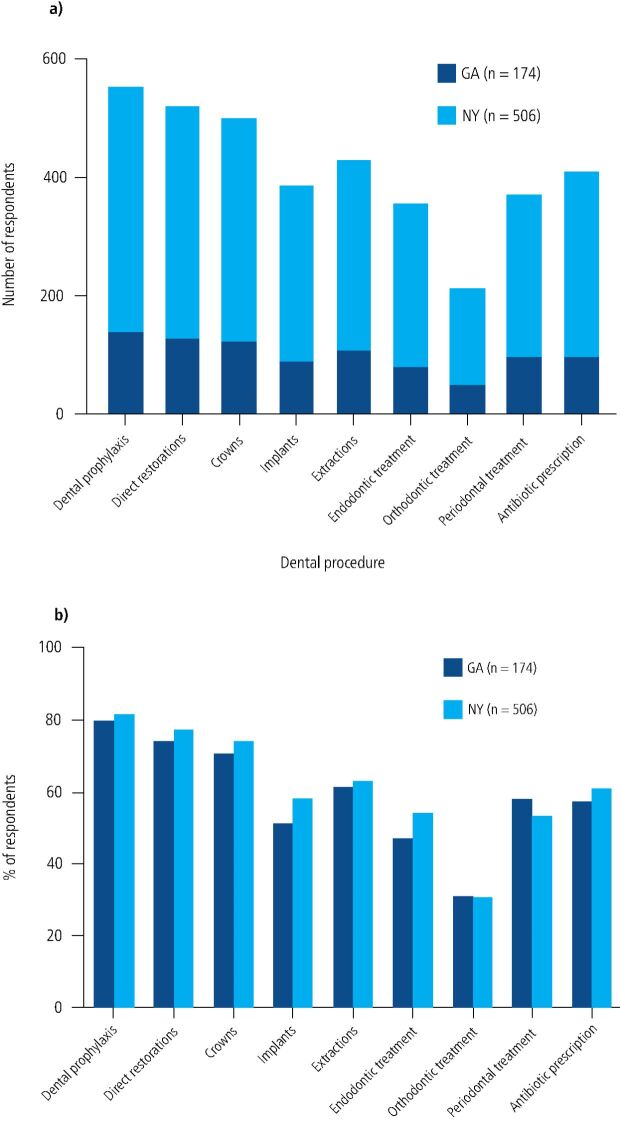
a) Number of respondents per procedure in Georgia versus New York. b) Percent respondents per procedure in Georgia versus New York. Due to differences in respondents' specialties, not all respondents answered all questions. There were no significant differences in percent respondents between New York State and Georgia

Statistical analysis of responses was conducted using statistical software embedded in Excel (Microsoft, Redmond, WA). No sample size calculation was performed due to the observational and descriptive nature of this study. Every available, qualified, survey response was included in our analysis of the provision of dental care in New York and Georgia. Comparisons between the two states also included all available, qualified survey data.Survey responses from zip codes outside NY and GA, from dentists that chose not to share their zip code and whose state could not be determined, and from retired dentists who were not in practice during the COVID-19 pandemic, were excluded from data analysis. Two-way t-tests were used to determine significant differences between the means of the NYSDA and GDA groups and chi-square tests were applied to assess differences between categorical variables. Both analyses set statistical significance at 0.05. Due to differences in respondents' specialties, not all respondents answered all questions (Fig. 1).

## Results

### Participant characteristics

The response rates of members of the NYSDA and GDA were remarkably similar, at 5.06% and 4.89%. Most respondents had been in dental practice for >20 years (range: <5 years to >20 years). Nearly half of respondents from both NYSDA and GDA reported practising in a solo dentist practice, with less than 3% of respondents in each association practising in offices shared by more than ten dentists (Table 1). Chi-squared analysis determined that time of reopening was not a function of years in practice, nor of the number of dentists in each practice. Medicaid was accepted by 15.81% of NYSDA respondents' practices and 17.24% of GDA respondents' practices (p = 0.51).

**Table 1:** Demographics of respondent sample. The majority of respondents in both New York and Georgia are solo practitioners that have been in practice for more than 20 years and do not accept Medicaid. There is no statistically significant difference in demographic characteristics of NYSDA and GDA respondents

Respondent demographics	(Total n = 680)	
**Years in practice**	**Number**	**Percentage %**
<5 years	34	5
5-10 years	61	9
11-20 years	109	16
>20 years	476	70
**Number of dentists practising in the respondents' practice**		
Solo dentist	353	52
2-9 dentists	306	45
10+ dentists	20	3
**Medicaid acceptance of respondents' dental practices**		
Accepts Medicaid	109	16
Does not accept Medicaid	571	84

### Reopening non-urgent dental care

**Fig. 2:**
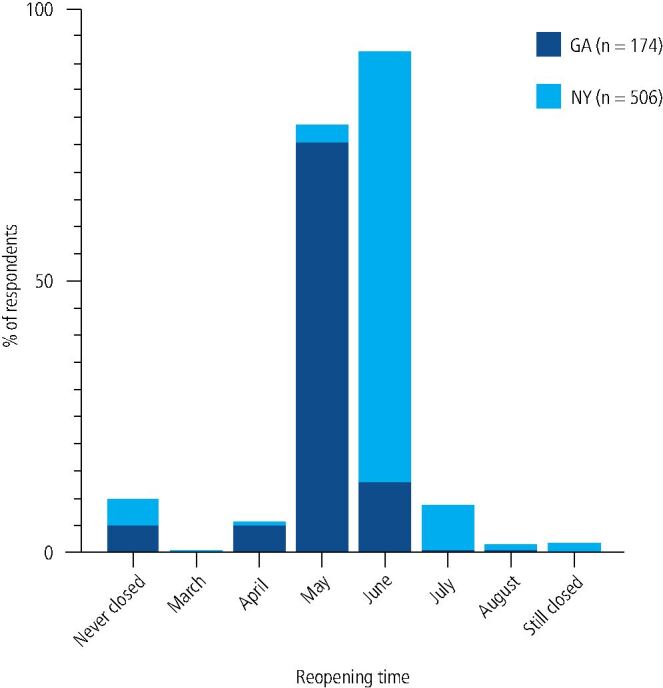
Dental practice full reopening times for NYSDA and GDA members. The study's results are largely consistent with the state mandates of New York and Georgia to reopen dental practices for non-urgent dental care on 1 June and 1 May, respectively

Georgia formally permitted the full reopening of dental offices on 1 May 2020 and 75.3% of Georgian dental respondents reopened their practice in May 2020 for non-urgent dental care (Fig. 2). However, 13.2% of surveyed Georgian dentists waited until June to reopen, and some reported opening before the permitted full reopening time or never fully closing their dental practice. Interestingly, a nearly identical proportion of NYSDA (4.95%) and GDA (5.17%) respondents stated that their dental practice was never closed during the COVID-19 pandemic. New York formally permitted the full reopening of dental offices on 1 June 2020 and 79% of New York dental respondents reported reopening their practices in June 2020 for non-urgent care. Like Georgia, 8.3% of New York dentists hesitated to reopen until July 2020, a month later. But while nearly 2% of surveyed New York dentists had not yet started accepting routine non-urgent procedures at the time of their survey response in September of 2020, all respondent Georgia dentists had reopened their practice for routine non-urgent procedures by that time.

### Appointment cancellations

The survey assessed COVID-19's impact on dental appointment cancellation rates and to what degree patients' fear of COVID-19 exposure factored into their practice's cancellations. Dentists' opinion of how the cancellation rate changed from March to August of 2020 was compared to the same five-month period in 2019. The change in cancellation rates showed a wide distribution, ranging from half as many cancellations compared to the year before to twice as many cancellations. As a result, the percent change for both states averaged closer to 0% change. Nonetheless, on average, New York and Georgia dental respondents felt cancellations were due to COVID-19 exposure fear 37% and 52% of the time, respectively.

### Dental procedures provided

**Fig. 3:**
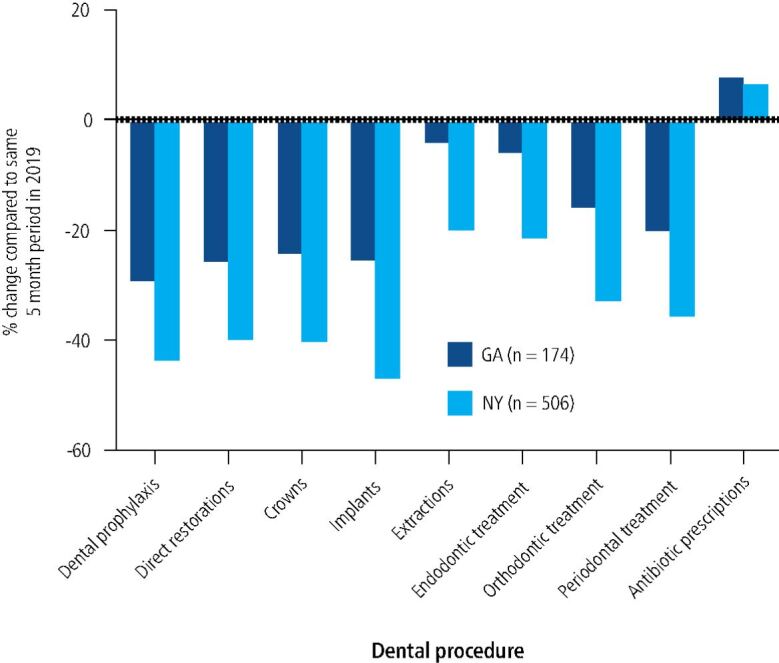
Results of survey question: 'from 1 March to 1 August 2020, compared to the same five-month period in 2019, how did the volume of each dental procedure provided to your patients change?'. NYSDA members reported significantly larger decreases in performing all types of dental procedures. Antibiotic prescription was the only dental care service whose change from pre-COVID baseline was not significantly different between New York and Georgia

The primary goal of this research was to quantify and document what specific dental procedures were provided relative to 'normal' volumes during the hiatus in dental care enforced during the COVID-19 pandemic from March to August 2020 (Fig. 3).

Dental prophylaxis during the COVID-19 pandemic from March to August 2020 decreased by 29% in Georgia and 44% in New York compared to the prior year. T-tests confirmed a statistically significant difference between New York and Georgia respondents (p <0.001).

New York dental respondents reported a decrease of 40% for direct restorations and crowns and a decrease of 47% for implants compared to the same timeframe in 2019. Georgia dental respondents reported a decrease of ~25% for direct restorations, crowns and implants alike. Each of these interstate comparisons were statistically significant for direct restorations (p <0.001), crowns (p <0.001) and implants (p <0.001).

Emergency procedures, including extractions and endodontic treatment, declined to a lesser extent across both states. New York dental respondents reported a decrease of 20% and 21% for extractions and endodontic treatment, respectively. Georgia dental respondents reported a decrease of 4% and 6% for extractions and endodontic treatment, respectively. Again, interstate comparisons were statistically significant for both extractions (p <0.001) and endodontic treatment (p <0.001).

Other specialty procedures generally considered slightly less urgent, including orthodontic and periodontal treatment, decreased to a greater extent across both states. New York dental respondents reported a decrease of 33% and 35% for orthodontic and periodontal treatment, respectively. Georgia dental respondents reported a decrease of 16% and 20% for orthodontic and periodontal treatment, respectively. P-values were significant for both interstate comparisons (p = 0.0011 for orthodontic treatment, p <0.001 for periodontal treatment).

Antibiotic prescription compared to 'normal' increased by 6.5% and 7.7% according to New York and Georgia dentists, respectively. Antibiotic prescription was the only dental care service whose change from baseline was not significantly different between New York State and Georgia (p = 0.75).

## Discussion

This study strives to quantify the individual dental procedures provided during the forced hiatus in dental care associated with the COVID-19 pandemic from March to August 2020 compared to the same period the year prior in the states of New York and Georgia.

The extent to which respondents to our questionnaire are representative of the general population of dentists is worthy of examination. The demographic finding that 70% of the respondents have been in practice for more than 20 years is consistent with the ADA's Health Policy Institute findings that the average American dentist's age was 49.3 in 2020.^9^ The typical age at which American dentists graduate is approximately 28 years.^10^ The ADA found that 'half of private practice dentists work solo', also consistent with 52% of this study's respondents reporting that they are a solo practitioners.^9^ While this study's results found 16% and 17% of NYSDA and GDA respondents' practices accept Medicaid, respectively, 36.5% of dentists in New York State participate in Medicaid while 27.5% of dentists in Georgia participate in Medicaid.^11^ It is possible that Medicaid-accepting dentists are less likely to become members of these dental associations, but because this information is not collected by either dental association, respondent bias cannot be ruled out.

Dentists in both states reported a statistically significant decrease in all dental procedures, in particular, dental prophylaxis, during the COVID-19 pandemic. These findings in the USA differ from the reported 83.8% of Brazilian dentists who reported that their patients sought out elective care during the pandemic.^8^ This contrast highlights the international inconsistencies in attitudes and management of the COVID-19 pandemic.

Despite NYSDA and GDA members having no statistically significant differences in the demographic makeup of respondents in this study, NYSDA members had a significantly larger decrease in prophylaxis, elective care, emergency dental care and speciality procedures.

Although state governors mandated the opening and closing of dental practices to non-urgent dental care, this research reveals that compliance with these mandates was imperfect in both New York and Georgia. Roughly 5% of respondents in both states responded that their dental practice was never closed for non-urgent dental care during the COVID-19 pandemic. Interestingly, 8.3% and 13.2% of New York and Georgia respondents, respectively, waited a month after permitted by state government to reopen their practice to elective dental care. A varied interpretation of 'emergency dental care' may have also contributed to these inconsistencies.

New York dentists were advised to reopen non-urgent dental care a month later than Georgia dentists, but the consistently greater decrease in dental care in New York over the five-month period suggests other external factors at play. These results might reflect the increased general fear and COVID-19 impact felt by citizens and dental practitioners in New York, and New York City in particular, during the Spring of 2020. New York City and secondly New York State held the highest cumulative number of reported COVID-19 cases, the highest cumulative incidence and the highest number of reported COVID-19-related deaths in the USA from 12 February to 7 April, while these metrics were much lower in Georgia.^12^ Such significant differences in the COVID-19 experience in New York State and Georgia may explain the consistently greater decline in dental care in New York. These findings may suggest an increased fear of reopening dental offices in New York for dentists and patients alike.

As the chi-squared analysis determined that time of reopening was not a function of years in practice, we can conclude that dentists' number of years of practice experience had no statistically significant impact on time of reopening in New York and Georgia. One could speculate that increased confidence gained through years of experience might have been counterbalanced by an increased fear of age-related COVID-19 morbidity and mortality among older, more experienced dentists. This finding differs from survey results in Brazil in which a higher percentage of younger dentists continued routine dental treatment with less concern compared to older dentists.^8^ Further, chi-squared analysis found that the number of dentists in a group practice also had no significant impact on time of reopening in this study.

The only dental procedure that did not significantly differ from baseline 2019 levels or between New York State and Georgia was antibiotic prescription. It might have been anticipated that New York dentists, who had a greater decrease in in-person care, would prescribe more antibiotics to treat patients remotely, but the findings of this survey demonstrated a similar increase in antibiotic prescription to Georgia dentists. The explanation remains unknown, but New York patients' readiness to reach out remotely for dental care due to COVID-19 fear could be a contributing factor.

### Study limitations

The survey response rate of 5.06% for NYSDA and 4.89% for GDA raises the possibility that respondents may differ from non-respondent dentists, diminishing the generalisability of these results. Correction and stratification of potential respondent bias could not be easily resolved, as the data were collected anonymously through the professional associations. Neither the New York State nor the Georgia Dental Association collects information on their membership's years in practice, number of dentists per practice, nor whether they accept Medicaid. Without this baseline demographic information, the extent to which respondent dentists represent the broader membership of the dental associations is unknown.

The survey sample might also be subject to recall bias, as all parties were asked to recount procedures and cancellations from months prior.

Finally, limitations may include erroneous responses to question six: 'how did the cancellation/rescheduling rate compare to your dental practice's usual rate when your office reopened after the COVID-19 dental hiatus?'. Question results were widely distributed and averaged close to 0% change, diminishing confidence in the interpretation by respondents, despite apparent success in the pilot testing. While NYSDA and GDA members felt COVID-19 exposure fear contributed to 37% and 52% of dental appointment cancellations, respectively, it might have been anticipated that the cancellation rate compared to normal would be a clear increase. It is possible that the dentists and their patients were more affected by other factors, although this cannot be determined from the present data.

This survey was conducted to better understand the impact of the unprecedented hiatus in dental care during the COVID-19 pandemic on oral public health. The sample groups of dentists from New York and Georgia who accepted the invitation to participate in this survey were similar in terms of years in practice, size of practice and acceptance of Medicaid.

Across the states of New York and Georgia, the provision of all dental procedures declined significantly during the COVID-19 pandemic. This decrease was significantly greater in New York than in Georgia, particularly with dental prophylaxis, direct restorations, crowns and implants.

## Conclusions

In brief, the deferral of dental care during the COVID-19 pandemic that this study quantified is likely to cause a decline in oral public health. Dentists throughout the world should actively encourage their patients to resume routine dental prophylaxis to mitigate against this risk, while maintaining optimal infection prevention and control measures to ensure the safety of their patients. It will be interesting to see if cities hit harder by the pandemic, who received significantly less dental care, will present with more advanced dental disease and worse dental prognosis in the aftermath of the COVID-19 pandemic. Future investigations can use this quantification of the dental care provided during the COVID-19 dental hiatus to assess the impact and repercussions of the COVID-19 pandemic on oral public health in the near and more distant future.

## Supplementary Information

Qualtrics Survey (PDF 81KB)

## References

[1] Lucaciu O, Tarczali D, Petrescu N. Oral healthcare during the COVID-19 pandemic. *J Dent Sci* 2020; **15:** 399-402. 10.1016/j.jds.2020.04.012 PMC7252092 32837682

[2] Innes N, Johnson I G, Al-Yaseen W *et al.* A systematic review of droplet and aerosol generation in dentistry. *J Dent* 2021; **105:** 103556. 10.1016/j.jdent.2020.103556 PMC7834118 33359043

[3] To K K-W, Tsang O T-Y, Yip C C-Y *et al.* Consistent Detection of 2019 Novel Coronavirus in Saliva. *Clin Infect Dis* 2020; **71:** 841-843. 10.1093/cid/ciaa149 PMC7108139 32047895

[4] COVIDental Collaboration Group. The COVID-19 pandemic and its global effects on dental practice. An International survey. *J Dent *2021; **114:** 103749. 10.1016/j.jdent.2021.103749 PMC8285212 34280498

[5] Centers for Disease Control and Prevention. Guidance for Dental Settings: Interim Infection Prevention and Control Guidance for Dental Settings During the COVID-19 Response. 2020. Available at https://dental.nv.gov/uploadedFiles/dentalnvgov/content/Home/06.17.20%20NSBDE%20CDC%20dental%20settings-PS%20markup.pdf (accessed January 2023).

[6] American Dental Association. COVID-19 State Mandates and Recommendations. 2020. Available at https://www.ada.org/ (accessed June 2020).

[7] Chang H-J, Huang N, Lee C-H, Hsu Y-J, Hsieh C-J, Chou Y-J. The impact of the SARS epidemic on the utilization of medical services: SARS and the fear of SARS. *Am J Public Health* 2004; **94:** 562-564. 10.2105/ajph.94.4.562 PMC1448298 15054005

[8] Faccini M, Ferruzzi F, Mori A A *et al.* Dental Care during COVID-19 Outbreak: A Web-Based Survey. *Eur J Dent* 2020; DOI: 10.1055/s-0040-1715990. 10.1055/s-0040-1715990 PMC7775249 32882738

[9] American Dental Association. The Dentist Workforce — Key Facts. 2021. Available at https://www.ada.org/ (accessed June 2020).

[10] American Dental Education Association. US Dental School Applicants and Enrollees, 2010 Entering Class. Available at bit.ly/3Wijgcp (accessed January 2023).

[11] American Dental Association. Geographic Access to Dental Care. Available at https://www.ada.org/ (accessed June 2020).

[12] CDC COVID-19 Response Team. Geographic Differences in COVID-19 Cases, Deaths, and Incidence - United States, February 12 - April 7, 2020. *MMWR Morb Mortal Wkly Rep* 2020; **69:** 465-471. 10.15585/mmwr.mm6915e4 PMC7755058 32298250

