# Supplementary material for: The impact of COVID-19 on dental care in New York State and Georgia
Source: Br Dent J. 2023 Jan 24:1–6. Online ahead of print. doi: 10.1038/s41415-023-5458-9 (PMC9873203; doi:10.1038/s41415-023-5458-9)
Supplement: Supplementary file 1 — Qualtrics Survey (PDF 81KB) [file 41415_2023_5458_MOESM1_ESM.pdf]

COVID-19's Impact on Dental Care in New York State and Georgia

---

Start of Block: Default Question Block

Q1 In what zip code is your primary office located?

____

Q2 How many years have you been in practice?

- ○ <5 years (1)
- ○ 5-10 years (2)
- ○ 11-20 years (3)
- ○ >20 years (4)

Q3 What is the size of your dental practice?

- ○ Solo dentist (1)
- ○ 2-9 dentists (2)
- ○ 10+ dentists (3)

Q4 Does your dental practice accept Medicaid?

- ○ Yes (1)
- ○ No (2)

Q5 When did your dental practice fully reopen and offer routine non-emergent procedures after the 2020 COVID-19 hiatus in dental care?

- ○ My dental practice was never closed (1)
- ○ March (2)
- ○ April (3)
- ○ May (4)
- ○ June (5)
- ○ July (6)
- ○ August (7)
- ○ My dental practice has yet to accept routine non-emergent procedures (8)

Q6 How did the cancellation/rescheduling rate compare to your dental practice's usual rate when your office reopened after the COVID-19 dental hiatus?

	100% decrease	No change	100% increase	Not Applicable
	-100 -75 -50 -25	0	25 50 75 100	
Cancellation/rescheduling rate ()				

Q7 Approximately what percentage of cancellations/rescheduled appointments do you suspect were due to patients' fear of COVID-19 exposure?

	0% of cancellations	50%	100% of cancellations	Not Applicable
	0 13 25 38	50	63 75 88 100	

Cancellations for fear of COVID-19 ()	

Q8 From March 1st to August 1st 2020 compared to the same 5 month period in 2019, how did the amount of **dental prophylaxis** provided to your patients change?

	100% decrease		No change		100% increase		Not Applicable		
	-100	-75	-50	-25	0	25	50	75	100
Dental prophylaxis ()									

Q9 From March 1st to August 1st 2020 compared to the same 5 month period in 2019, how did the amount of **direct restorations** provided to your patients change?

	100% decrease		No change		100% increase		Not Applicable		
	-100	-75	-50	-25	0	25	50	75	100
Direct restorations ()									

Q10 From March 1st to August 1st 2020 compared to the same 5 month period in 2019, how did the amount of **crowns** provided to your patients change?

	100% decrease		No change		100% increase		Not Applicable		
	-100	-75	-50	-25	0	25	50	75	100
Crowns ()									

Q11 From March 1st to August 1st 2020 compared to the same 5 month period in 2019, how did the amount of **implants** provided to your patients change?

	100% decrease		No change		100% increase		Not Applicable	
	-100 -75	-50	-25	0	25	50	75	100
Implants ()								

Q12 From March 1st to August 1st 2020 compared to the same 5 month period in 2019, how did the amount of **extractions** provided to your patients change?

	100% decrease		No change		100% increase		Not Applicable	
	-100 -75	-50	-25	0	25	50	75	100
Extractions ()								

Q13 From March 1st to August 1st 2020 compared to the same 5 month period in 2019, how did the amount of **endodontic treatment** provided to your patients change?

	100% decrease		No change		100% increase		Not Applicable	
	-100 -75	-50	-25	0	25	50	75	100
Endodontic treatment ()								

Q14 From March 1st to August 1st 2020 compared to the same 5 month period in 2019, how did the amount of **orthodontic treatment** provided to your patients change?

100% decrease | No change | 100% increase | Not Applicable

	-100	-75	-50	-25	0	25	50	75	100
Orthodontic treatment ()									

Q15 From March 1st to August 1st 2020 compared to the same 5 month period in 2019, how did the amount of **periodontal treatment** provided to your patients change?

	100% decrease	No change	100% increase	Not Applicable

	-100	-75	-50	-25	0	25	50	75	100
Periodontal treatment ()									

Q16 From March 1st to August 1st 2020 compared to the same 5 month period in 2019, how did the amount of **antibiotic prescriptions** provided to your patients change?

	100% decrease	No change	100% increase	Not Applicable

	-100	-75	-50	-25	0	25	50	75	100
Antibiotic prescriptions ()									

End of Block: Default Question Block
